# Nitroxoline impairs tumor progression *in vitro* and *in vivo* by regulating cathepsin B activity

**DOI:** 10.18632/oncotarget.3699

**Published:** 2015-03-30

**Authors:** Bojana Mirković, Boštjan Markelc, Miha Butinar, Ana Mitrović, Izidor Sosič, Stanislav Gobec, Olga Vasiljeva, Boris Turk, Maja Čemažar, Gregor Serša, Janko Kos

**Affiliations:** ^1^ Faculty of Pharmacy, University of Ljubljana, Ljubljana, Slovenia; ^2^ Department of Experimental Oncology, Institute of Oncology Ljubljana, Ljubljana, Slovenia; ^3^ Department of Biochemistry and Molecular and Structural Biology, Jožef Stefan Institute, Ljubljana, Slovenia; ^4^ Centre of Excellence for Integrated Approaches in Chemistry and Biology of Proteins, Ljubljana, Slovenia; ^5^ Faculty of Chemistry and Chemical Technology, University of Ljubljana, Ljubljana, Slovenia; ^6^ Department of Biotechnology, Jožef Stefan Institute, Ljubljana, Slovenia

**Keywords:** nitroxoline, cathepsin B, tumor invasion, angiogenesis, metastasis

## Abstract

Cathepsin B is a ubiquitously expressed lysosomal cysteine protease that participates in protein turnover within lysosomes. However, its protein and activity levels have been shown to be increased in cancer. Cathepsin B endopeptidase activity is involved in the degradation of extracellular matrix, a process that promotes tumor invasion, metastasis and angiogenesis. Previously, we reported an established antibiotic nitroxoline as a potent and selective inhibitor of cathepsin B. In the present study, we elucidated its anti-tumor properties in *in vitro* and *in vivo* tumor models.

Tumor and endothelial cell lines with high levels of active cathepsin B were selected for functional analysis of nitroxoline *in vitro*. Nitroxoline significantly reduced extracellular DQ-collagen IV degradation by all evaluated cancer cell lines using spectrofluorimetry. Nitroxoline also markedly decreased tumor cell invasion monitored in real time and reduced the invasive growth of multicellular tumor spheroids, used as a 3D *in vitro* model of tumor invasion. Additionally, endothelial tube formation was significantly reduced by nitroxoline in an *in vitro* angiogenesis assay. Finally, nitroxoline significantly abrogated tumor growth, angiogenesis and metastasis *in vivo* in LPB fibrosarcoma and MMTV-PyMT breast cancer mouse models. Overall, our results designate nitroxoline as a promising drug candidate for anti-cancer treatment.

## INTRODUCTION

Cathepsin B (CatB; EC 3.4.22.1) is a lysosomal cysteine protease that belongs to the papain family (C1) of clan CA of cysteine proteases. The enzyme possesses endopeptidase and dipeptidyl carboxypeptidase activity. This dual character is attributed to the presence of a 20 amino acid insertion termed the occluding loop. In the exopeptidase conformation, two salt bridges bind the loop to the body of the enzyme, in this way limiting the access of extended substrates to the primed sites of the active-site cleft [1]. The His110-His111 pair is located at the tip of the occluding loop and acts as an anchor for the substrate's C-terminal carboxylate. This enables the exopeptidase activity with a pH optimum around 5, which is typical for lysosomal compartments [2, 3]. However, the loop is flexible and removal of the contacts that bind it to the enzyme body markedly increases the endopeptidase activity. The endopeptidase conformation of the occluding loop is stable at neutral pH [4, 5], suggesting an endopeptidase activity for CatB in the extralysosomal and extracellular environment.

CatB primarily partakes in protein turnover in lysosomes; however, its physiological roles were recently extended to more specific functions. Alterations in its expression, protein levels, activity and localization are associated with several diseases, including cancer [6-8]. Intracellular and extracellular CatB were shown to degrade several proteins of the extracellular matrix – collagen type IV, laminin and fibronectin and activate proteases acting downstream in a proteolytic cascade resulting in extracellular matrix (ECM) degradation, tumor invasion and metastasis [9-13].

The active role of CatB in malignant progression was demonstrated in various tumor mouse models using CatB-deficient mice [14, 15]. Given the high pharmacological relevance of CatB in cancer, searching for new selective inhibitors became a challenge for medicinal chemists and pharmaceutical companies [16, 17]. The majority of synthetic inhibitors of CatB have peptidyl backbones with an electrophilic reactive group that forms either a reversible or an irreversible covalent bond with the active site cysteine. However, none of the existing CatB inhibitors are used in clinical practice due to poor bioavailability, off-target side effects and high toxicity [18]. Recently, we identified nitroxoline (5-nitro-8-hydroxyquinoline) as a potent, selective and reversible inhibitor of CatB [19]. Nitroxoline is an established drug for treatment of urinary tract infections and as such it displays superior pharmacokinetic/pharmacodynamic characteristics over existing CatB inhibitors. Independently, nitroxoline was also found to induce senescence of endothelial cells by inhibiting type 2 methionine aminopeptidase (MetAP2) and sirtuin (SIRT1) [20] and to induce cell-cycle arrest and apoptosis in glioma cells [21].

In the current study we evaluated nitroxoline's potential to impair tumor-promoting processes that depend on the endopeptidase activity of CatB. We demonstrate that inhibition of CatB endopeptidase activity by nitroxoline significantly reduces degradation of ECM and consequently the invasion of selected tumor cells in two-dimensional (2D) and three-dimensional (3D) models. Nitroxoline also impairs endothelial cell tube formation and significantly abrogates tumor growth and metastasis formation *in vivo*. These results encourage further pre-clinical and clinical studies to validate the potential of nitroxoline and its derivatives as promising antitumor drugs.

## RESULTS

### CatB protein and activity levels

The cell lines selected for *in vitro* evaluation of tumor cell invasion, metastasis and angiogenesis were of either human (MCF-10A neoT, U-87 MG, HUVEC and HMEC-1) or mouse (MMTV-PyMT, LPB and SVEC4-10) origin and comprised a variety of cancer types (transformed breast epithelial cell line MCF-10A neoT, mammary carcinoma cell line MMTV-PyMT, glioma cell line U-87 MG and sarcoma cell line LPB) as well as a variety of vascular cell lines of different origins (microvascular endothelial cell line HMEC-1 and vein endothelial cell lines HUVEC and SVEC4-10). Our first objective was to determine the CatB protein and activity levels associated with these cell lines.

**Figure 1:**
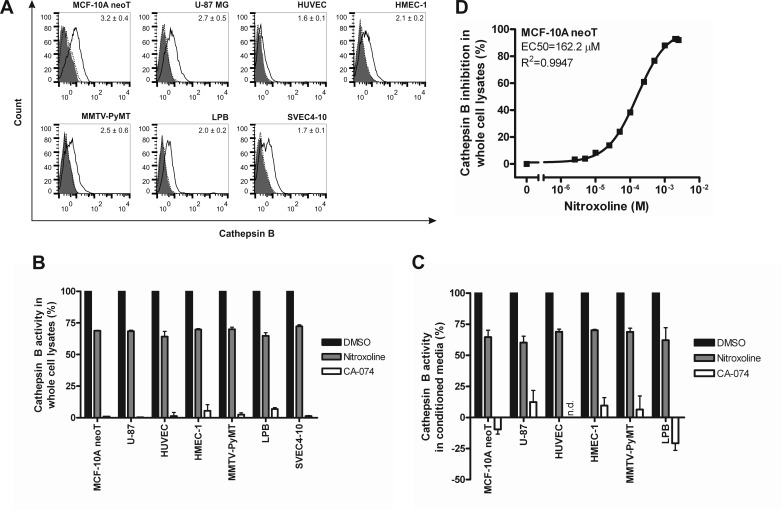
Cathepsin B cell surface expression and inhibition of its activity in whole cell lysates and conditioned media (**A**) All evaluated cell lines stained positive for CatB at the cell surface as shown with flow cytometry. Inserted values denote the fold increase in mean fluorescence intensity of cells stained for CatB (black line) compared to cells stained only with the secondary antibody (dotted black line). Solid grey histograms represent unlabeled cells. (**B**) Nitroxoline (100 μM) and CA-074 (10 μM) reduce CatB activity in whole cell lysates and (**C**) conditioned media as evaluated with CatB substrate Z-Arg-Arg-AMC. (**D**) Analysis of dose-response inhibition of nitroxoline on the activity of CatB in MCF-10A neoT lysates. All data are presented as means±SEM (n=3); n.d., not determined.

All cell lines were shown to contain a significant amount of CatB within the cell (Table 1) and bound to the extracellular surface of the plasma membrane (Fig. 1A) using CatB-specific ELISA and flow cytometry. Association of CatB with the plasma membrane was also confirmed with confocal microscopy (Supplementary Fig. 1). In addition, secreted CatB was observed for all cell lines apart from SVEC4-10 (Table 1). CatB protein and activity levels in cell lysates were significantly higher than those in conditioned media for all cell lines tested. In line with previous reports [22-24], human transformed and tumor cell lines, MCF-10A neoT and U-87 MG, had higher levels of intracellular and plasma membrane bound CatB than non-tumor vascular endothelial cell lines (p<0.001 and p<0.05, respectively) (Table 1 and Fig. 1A). However, this trend was not apparent in the murine cell lines.

**Table 1:** CatB protein and activity levels in whole cell lysates and conditioned media Data are presented as means±STDEV, n ≥ 3

	CatB protein levels(ng/mg total protein)		CatB activity levels(RFUs^−1^/mg total protein)	
	Whole cell lysates	Conditioned media	Whole cell lysates	Conditioned media
Human cell lines					
MCF-10A neoT	508±55	3.4±0.3	574±42	0.45±0.04
U-87 MG	602±72	1.1±0.1	616±99	0.31±0.03
HUVEC	82±14	0.5±0.1	168±24	0.13±0.01
HMEC-1	266±14	1.3±0.4	379±30	0.14±0.03
Mouse cell lines					
MMTV-PyMT	29±5	0.3±0.1	183±29	0.03±0.01
LPB	28±4	0.2±0.0	104±9	0.08±0.02
SVEC4-10	30±6	n.d.	90±16	0.01±0.00

n.d. Not detectable

CatB substrate Z-Arg-Arg-7-amino-4-methylcoumarin (AMC) was used to establish that CatB, regardless of its location, is proteolytically active (Table 1). Similar trends in CatB activity were observed as with CatB protein levels, *i.e.* levels of CatB activity in human transformed and cancer cell lines were higher than in human vascular endothelial cell lines (*p* < 0.001) and higher in human than in murine cell lines (*p* < 0.001). Irreversible CatB-selective inhibitor CA-074 (10 μM) [25] and nitroxoline (100 μM) inhibited the release of AMC in whole cell lysates and in conditioned media in all cell lines evaluated by ∼100 and ∼30%, respectively (Fig. 1B and 1C). Additionally, a half maximal effective concentration (EC50) was determined for nitroxoline inhibition of CatB activity in MCF-10A neoT whole cell lysates (162.2 μM; Fig. 1D). Taken altogether, these results validated the selected cell lines as suitable *in vitro* invasion and angiogenesis cell-based models for evaluation of CatB inhibitors.

### Nitroxoline reduces DQ-collagen IV degradation

**Figure 2:**
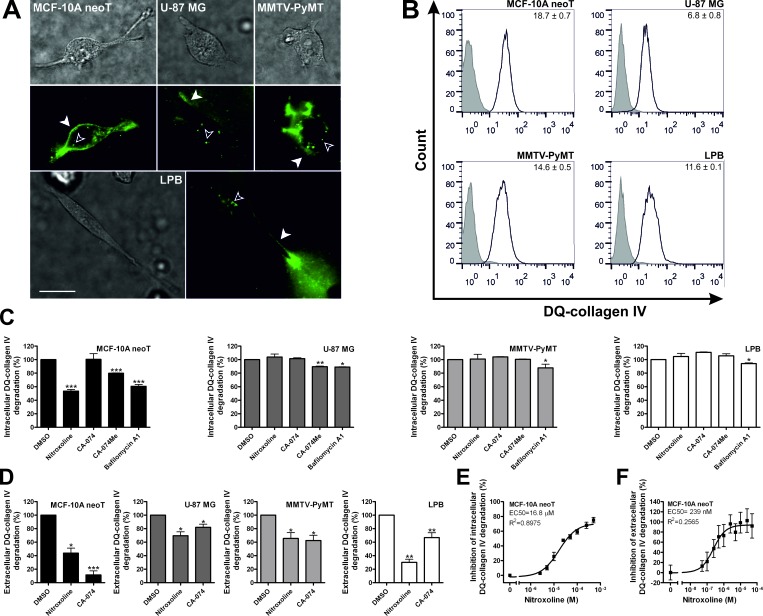
Nitroxoline impairs DQ-collagen IV degradation in transformed and tumor cells (**A**) 2.4×10^4^ MCF-10A neoT, U-87 MG, MMTV-PyMT and LPB cells were plated in wells of Lab-Tek™ Chambered Coverglass coated with 25 μg/ml DQ-collagen IV in 100% Matrigel. After 24h, the samples were monitored for degradation products using fluorescence microscopy. MCF-10A neoT, U-87 MG, MMTV-PyMT and LPB cells displayed intracellular (empty arrowheads) and extracellular (filled arrowheads) DQ-collagen IV degradation detected as green fluorescence. Upper panels are transmission images and lower panels are images of green fluorescence following hydrolysis of DQ-collagen IV. (**B**) Intracellular DQ-collagen IV degradation was further quantified with flow cytometry. Values, shown on individual histograms, present fold increase in mean fluorescence intensity after treatment of cells with DQ-collagen IV (5 μg/ml; black line) compared to non-treated cells (solid grey histograms). (**C**) Following treatment with nitroxoline (50 μM), CA-074 (50 μM), CA-074Me (50 μM) and bafilomycin A1 (100 nM) cells were monitored for reduction in intracellular DQ-collagen IV (5 μg/ml) degradation. (**D**) Next, extracellular DQ-collagen IV (10 μg/ml) degradation and its inhibition by nitroxoline (5 μM) and CA-074 (5 μM) were measured by spectrofluorimetry. Analysis of dose-response inhibition of nitroxoline on (**E**) intracellular and (**F**) extracellular DQ-collagen IV degradation by MCF-10A neoT cells. Data are presented as means±SEM, n=3 (*p < 0.05, **p < 0.01, ***p < 0.001). Scale bar, 20 μm.

Collagen IV is a major component of basement membrane that can be labeled with fluorescein, thus giving rise to bright green fluorescence upon proteolysis. MCF-10A neoT, U-87 MG, MMTV-PyMT and LPB cells all displayed intracellular and extracellular DQ-collagen IV degradation, as shown with fluorescence microscopy (Fig. 2A) and flow cytometry (Fig. 2B). CatB significantly contributes to intracellular and extracellular DQ-collagen IV degradation in cancer cells as shown by CatB knockdown (Supplementary Fig. 2). Pretreatment of MCF-10A neoT cells with nitroxoline (50 μM) or CA-074Me (50 μM), a cell-permeable CatB inhibitor, reduced intracellular DQ-collagen IV degradation by ∼50 and ∼20%, respectively (Fig. 2C). In contrast, CA-074 (50 μM), a non-permeable CatB inhibitor failed to impair intracellular DQ-collagen IV degradation. Bafilomycin A1 (100 nM), an inhibitor of vacuolar H^+^ ATPase that inhibits the acidification of lysosomes, reduced intracellular DQ-collagen IV degradation by ∼40%, suggesting that the degradation occurs within lysosomes and is dependent on lysosomal proteases. CA-074Me and bafilomycin A1, but not nitroxoline, inhibited intracellular collagen IV degradation in the U-87 MG glioma cell line by 10 and 11%, respectively (Fig. 2C). When murine MMTV-PyMT and LPB cell lines were evaluated, only bafilomycin A1 reduced intracellular DQ-collagen IV degradation, by 12 and 6% respectively, whereas no decrease was observed with nitroxoline or CA-074Me (Fig. 2C).

On the other hand, when collagen IV degradation was monitored in the extracellular milieu, far greater inhibition was evident. Nitroxoline (5 μM) and CA-074 (5 μM) inhibited extracellular DQ-collagen IV degradation in all tested cell lines (Fig. 2D). This was further corroborated by nitroxoline EC50 values (16.8 and 0.239 μM, respectively) for inhibition of intracellular and extracellular DQ-collagen IV degradation by MCF-10A neoT cells (Fig. 2E and 2F).

### Nitroxoline impairs *in vitro* tumor cell invasion in real time

**Figure 3:**
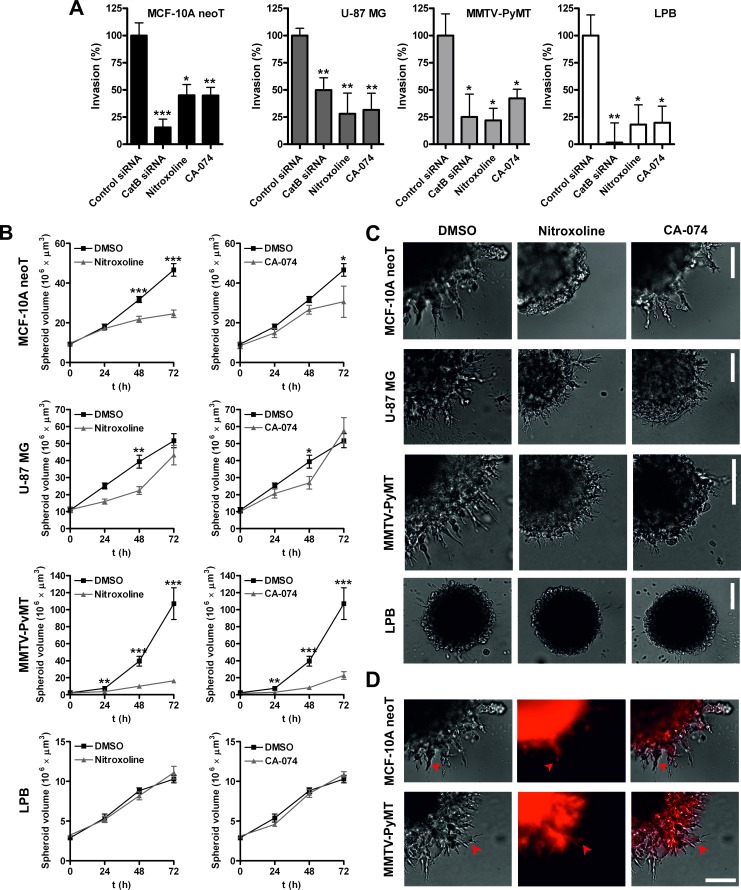
Nitroxoline impairs invasion of transformed and tumor cells (**A**) Invasion of cells was assessed in real time using the xCELLigence System [30]. siRNA-mediated silencing was performed 6h before the start of experiment using siRNAs targeting human or mouse CatB and control siRNA. Cells were also treated with nitroxoline (2.5 or 5 μM) and CA-074 (5 μM) or 0.05% DMSO as control and tumor cell invasion was monitored for inhibition in real time. (**B**) Next, MCF-10A neoT, U-87 MG, MMTV-PyMT and LPB MCTS were prepared according to the hanging-drop method [44] and implanted in Matrigel (5 mg/ml) which was covered with 400 μl of medium. Nitroxoline (2.5 or 5 μM), CA-074 (5 μM) or DMSO (0.05%) were added to Matrigel and medium. MCTS volume was monitored up to three days and (**C**) representative images of MCTS were obtained at day 2 (U-87 MG) and 3 (MCF-10A neoT, MMTV-PyMT and LPB) after implantation. (**D**) CatB activity in MCF-10A neoT and MMTV-PyMT spheroids was visualized using the Magic Red^TM^ probe and fluorescence microscopy. Left panels are transmission images, middle panels are red fluorescence images following hydrolysis of the probe and right panels represent merged images. Red arrow heads point to CatB activity in cells that actively invade the Matrigel surrounding the spheroid. Data are presented as means±SEM, n > 3 (*p < 0.05, **p < 0.01, ***p < 0.001). Scale bar, 100 μm.

To confirm the involvement of CatB in the invasion of the transformed and cancer cells small interfering RNA (siRNA)-mediated silencing was performed. CatB knockdown reduced invasion of all cell lines when compared to cells transfected with control siRNA, thus validating CatB as a tumor cell invasion-promoting protease (Fig. 3A). To verify the silencing, transfected cells were evaluated for CatB protein levels and activity at 24, 48 and 72h after the transfection (Supplementary Fig. 3 and 4).

Next, we evaluated the ability of nitroxoline to impair tumor cell invasion in real time using the xCELLigence System. As shown in Fig. 3A, nitroxoline (2.5 or 5 μM) reduced cell invasion in all the cell lines (54-93%), with the largest decrease in LPB cells. Similarly, CA-074 (5 μM) reduced invasion of all cell lines evaluated (47-68 %).

To exclude the possibility that the inhibition of tumor invasion resulted from the compound-induced cell toxicity, cell viability was assayed using the xCELLigence system. Neither nitroxoline (2.5 or 5 μM) nor CA-074 (5 μM) reduced the viability of cells at concentrations and times used for the invasion experiment (Supplementary Fig. 5).

Additionally, to explore the probability that nitroxoline decreased tumor cell invasion by reducing CatB expression, CatB protein levels were evaluated in cell lysates following treatment with nitroxoline. As depicted in Supplementary Fig. 6, nitroxoline (2.5 or 5 μM) did not significantly reduce CatB protein levels after 24, 48 and 72 h treatment as analyzed with western blot and ELISA, confirming that nitroxoline impairs tumor cell invasion by CatB inhibition.

### Nitroxoline inhibits the growth of multicellular tumor spheroids in a 3D *in vitro* tumor invasion assay

An *in vitro* invasion model based on implantation of multicellular tumor spheroids (MCTS) in ECM-mimicking matrices was used to assess the ability of nitroxoline to impair tumor cell invasion in a 3D setting [26]. Cell numbers and time of incubation needed for the preparation of MCTS of reproducible shape and size were optimized for each cell line individually (Supplementary Fig. 7). MCTS were implanted in Matrigel and their growth monitored for three days (Fig. 3B). Representative images were recorded at day 2 (U-87 MG) or day 3 (MCF-10A neoT, MMTV-PyMT and LPB) (Fig. 3C). Cells at the border of the MCF-10A neoT, U-87 MG and MMTV-PyMT MCTS formed multicellular, radially invading strands, creating a sunburst pattern around the original spheroid. In addition to collective invasion, individual cells were also observed migrating away from the original MCTS (Supplementary Fig. 8). Of the four cell lines used only LPB MCTS were without an invasive corona, but they nevertheless retained the individual cell invasive pattern (Fig. 3C). Abundant CatB proteolytic activity was observed in cells located in the core as well as at the protrusive edges of MCTS, using the CatB-selective activity probe cresyl violet-(Arg-Arg)_2_ (Magic Red^TM^) (Fig. 3D).

Incubation of MCF-10A neoT and MMTV-PyMT MCTS with nitroxoline (2.5 or 5 μM) or CA-074 (5 μM) reduced the spheroid growth (Fig. 3B) as well as the formation of invasive protrusions at the edge of MCTS (Fig. 3C). In addition, fewer cells were observed migrating away from the original spheroid after treatment with nitroxoline (Fig. 3C). A less pronounced impact was observed with U-87 MG MCTS, where significant inhibition of MCTS growth was observed at day 2 with reduced, albeit not significant, inhibition at day 3. However, as before, nitroxoline or CA-074 reduced corona sprouting and migration of individual cells. Nitroxoline or CA-074 failed to inhibit spheroid growth with LPB MCTS, but fewer cells migrated away from the original spheroid after treatment with nitroxoline (Fig. 3C).

### Nitroxoline impairs endothelial cell tube formation *in vitro*

**Figure 4:**
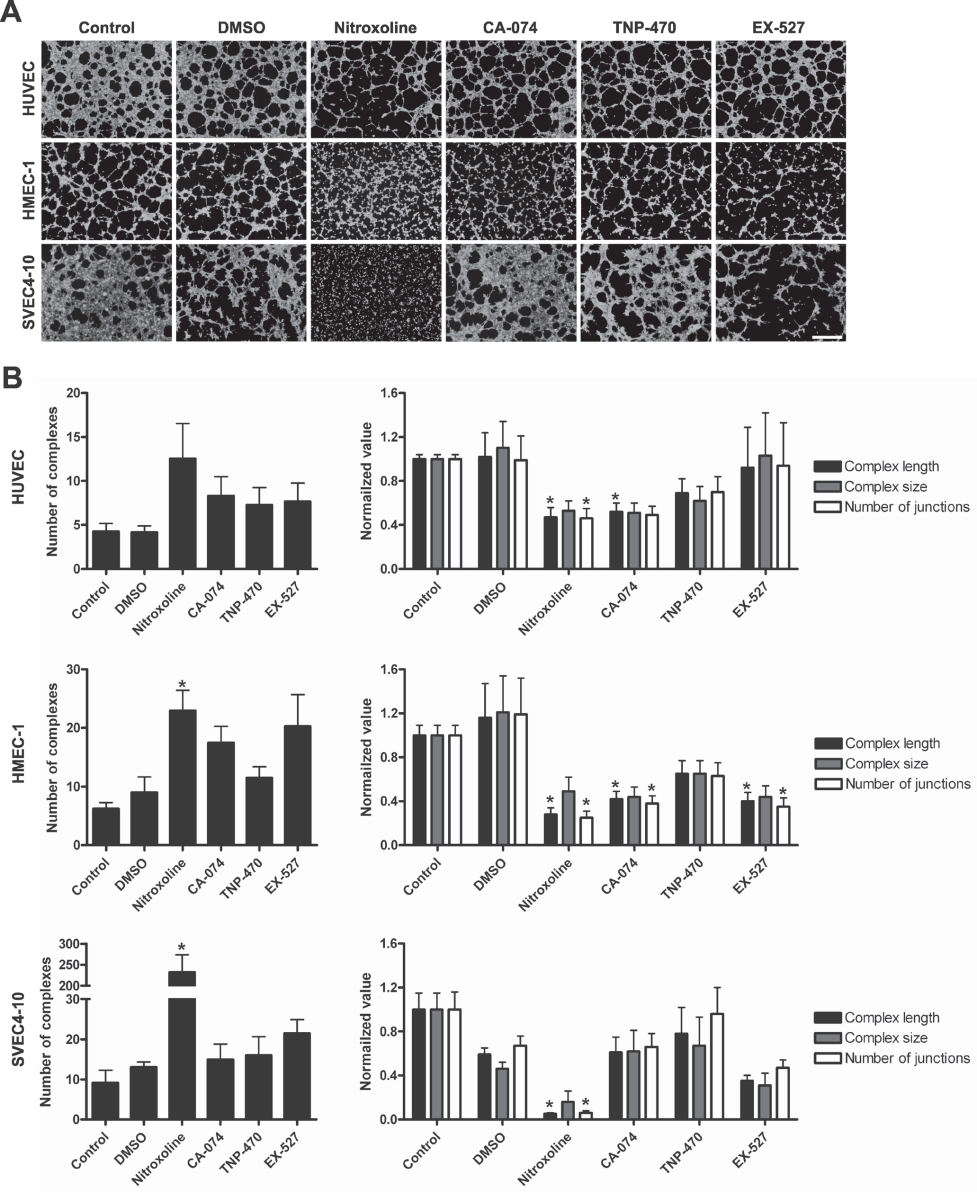
Treatment with nitroxoline impairs endothelial cell tube formation (**A**) Cells were incubated for 24h with nitroxoline (10 μM), CA-074 (10 μM), TNP-470 (10 nM) and EX-527 (1 μM) and thereafter plated on μ-Slide Angiogenesis covered with undiluted Matrigel and incubated until tubular complexes formed. These were visualized with Calcein AM and images of green fluorescence were captured with a fluorescent microscope. (**B**) AngioQuant image analysis program was used to quantify the number of tubular complexes as well as their length, size and the number of their junctions. Data are presented as means±SEM, n > 3 (*p < 0.05). Scale bar, 500 μm.

To determine the anti-angiogenic potential of nitroxoline an *in vitro* angiogenesis assay was performed. The assay employs a combination of fluorescence microscopy and analysis of binary masks of tubular networks created from original images. It quantifies the number of tubular complexes, their length and size that correlate with the capacity of endothelial cells to form new vessels. Nitroxoline (10 μM) reduced tube formation of HMEC-1 and SVEC4-10 cells as evident from increased numbers of tubular complexes, but not of HUVEC cells (Fig. 4A and 4B). Inhibitors of CatB, MetAP2 and SIRT1, CA-074 (10 μM), TNP-470 (10 nM) and EX-527 (1 μM), alone did not change the number of tubular complexes in all tested endothelial cell lines (Fig. 4B). Additional analysis of binary masks of tubular networks showed that nitroxoline also caused a reduction in the length of tubular complexes and in the numbers of their junctions in HUVEC, HMEC-1 and SVEC4-10 cells, without reducing their size. CA-074 also decreased the length of tubular complexes in HUVEC and HMEC-1 cell lines and the number of junctions in HMEC-1 cell line, but did not impair SVEC4-10 endothelial cell tube formation. TNP-470 failed to reduce the length and size of tubular complexes or the number of their junctions in any of the cell lines tested, whereas EX-527 did not impair HUVEC or SVEC4-10 endothelial tube formation, but reduced the complex length and the number of junctions of HMEC-1 cell line.

### Nitroxoline abrogates tumor growth *in vivo*

**Figure 5:**
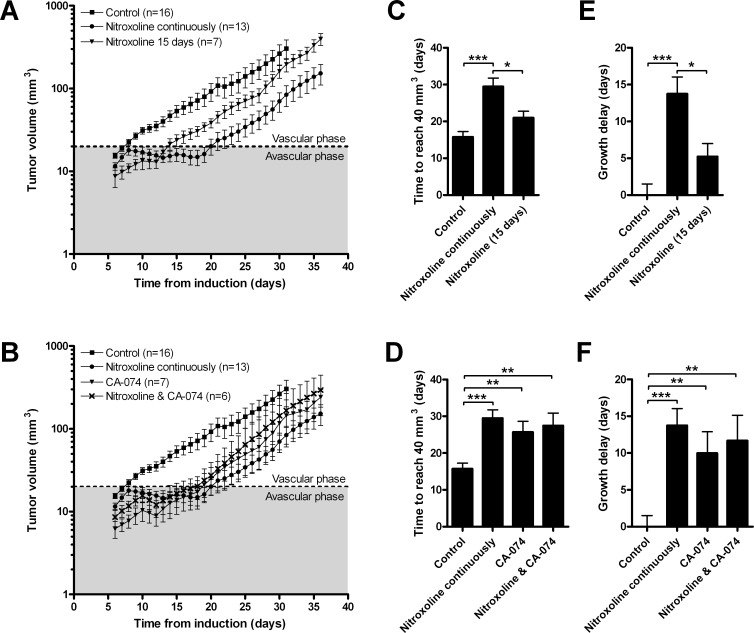
Nitroxoline reduces tumor growth in a LPB mouse tumor model LPB cells (1.2×10^6^) were injected into the right flank of C57Bl/6 mice. Mice were then treated with (**A**) nitroxoline (40 mg/kg, *ad libitum* in drinking water) for the entire duration of the study or 15 days only; (**B**) with CA-074 (10 mg/kg, intraperitoneally) every second day alone or in combination with nitroxoline and tumor growth of LPB-induced tumors was monitored every second day. Control group was exposed to water only. Growth curves were used to determine (**C** and **D**) the time required for tumors to reach a volume of 40 mm^3^, and (**E** and **F**) tumor growth delay, defined as the difference in the time required for tumors to reach a volume of 40 mm^3^ between the experimental and control group. Data are presented as means±SEM (*p < 0.05, **p < 0.01, ***p< 0.001 as determined with one-way ANOVA).

LPB fibrosarcoma cells were implanted in C57Bl/6 mice and nitroxoline (40 mg/kg) was administered in their drinking water for the entire duration of experiment or for 15 days only. As control, CA-074 (10 mg/kg) was administered intraperitoneally every second day, alone or in combination with nitroxoline in drinking water. Nitroxoline (Fig. 5A) as well as CA-074 (Fig. 5B) increased the time required for LPB tumors to reach a volume of 40 mm^3^ (Fig. 5C and 5D) and caused a delay in tumor growth (Fig. 5E and 5F). The reduced rate of tumor growth was observed at the transition of tumors from the avascular to the vascular phase of growth. Administration of CA-074 and nitroxoline in combination did not result in further reduction of tumor growth (Fig. 5B). When nitroxoline was withdrawn after 15 days of treatment, the nitroxoline-induced inhibition of tumor growth ceased (Fig. 5A).

**Figure 6:**
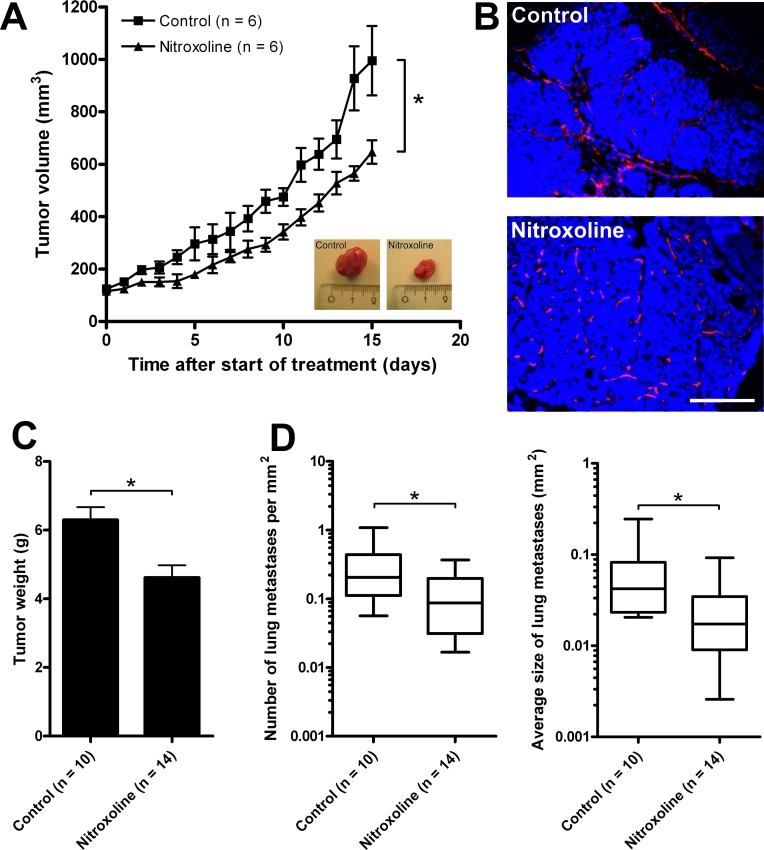
Nitroxoline attenuates tumor growth, metastasis and angiogenesis in MMTV-PyMT mouse breast cancer model (**A**) MMTV-PyMT cells (5×10^5^) were inoculated into the left inguinal mammary gland of the FVB/N congenic recipient mouse and after tumor volumes reached 125 mm^3^, mice were treated with 40 mg/kg nitroxoline intraperitoneally every second day. Control group was injected with vehicle only. Tumor volume was monitored daily and after conclusion of the experiment, tumors were excised (see inlet for images of representative tumors) and (**B**) vascularization of excised tumors was visualized with CD31 staining (red fluorescence). (**C**) In a separate experiment transgenic 11 weeks old PyMT mice were treated with 40 mg/kg nitroxoline every second day. Control group was injected with vehicle only. At 14 weeks of age mice were sacrificed and tumors collected and weighed. (**D**) Lungs were dissected and processed for volumetric measurement of total lung metastasis. 5 μm thick lung slices were stained with haematoxylin and eosin and the number and the average size of metastases was determined. Data are presented as means±SEM (*p < 0.05). Scale bar, 100 μm.

An orthotopic mouse breast cancer model was also employed in which primary MMTV-PyMT cells were injected into the left inguinal mammary gland of FVB/N congenic recipient mice. Nitroxoline (40 mg/kg) treatment of these mice reduced the growth of induced tumors (Fig. 6A). Neovascularization in treated tumors was examined by immunohistochemical staining for CD31+ endothelial cells. As shown in Fig. 6B nitroxoline impaired neovascularization of induced tumors and images obtained resembled the results from the *in vitro* tube formation assay, where increase in the number of tubular complexes was observed (Fig. 4).

The effect of nitroxoline (40 mg/kg) on the metastatic process was examined using the transgenic PyMT mouse model that spontaneously develops numerous mammary tumors and lung metastases [27]. Since the large number of spontaneously forming tumors prevented monitoring of the tumor volumes by palpation and Vernier caliper, the tumors were weighed at the end of the experiment. The weight of tumors at week 14 following nitroxoline treatment was significantly lower than that of tumors from untreated animals (Fig. 6C). Furthermore, in the nitroxoline-treated PyMT mice the number of lung metastases as well as their average size were reduced (Fig. 6D).

Nitroxoline did not induce any systemic toxicity as judged by monitoring the body weight of mice in all three *in vivo* tumor models (data not shown).

## DISCUSSION

Nitroxoline, an established antibiotic for treating urinary tract infections, has been shown to be a selective and reversible inhibitor of CatB endopeptidase activity which is involved in the degradation of ECM proteins [9, 10]. In this study we demonstrate that nitroxoline impairs various processes of tumor progression by reducing CatB activity in various *in vitro* assays using transformed and cancer cell lines and *in vivo* in three different tumor mouse models.

Our first aim was to select cell lines with appropriate expression, activity and secretion of CatB to establish *in vitro* invasion and angiogenesis models for evaluation of the CatB inhibitors. Significant levels of proteolytically active CatB were demonstrated in cell lysates, extracellular milieu and on the cell surface of all selected cell lines (Table 1 and Fig. 1A). Nitroxoline and CA-074 inhibited CatB activity in cell lysates and conditioned media by ∼30-40 and ∼100%, respectively. The different degrees of inhibition can be attributed to their mechanisms of action – while CA-074 irreversibly inactivates the enzyme in the nano-molar range [25], nitroxoline reversibly inhibits CatB at low micro-molar concentration [19]. Irreversible inhibitors may at higher doses and prolonged use cause undesired effects due to off-target binding rendering them inappropriate for therapy of chronic diseases [18]. Because of this, reversible inhibitors, although less efficient, may be superior to irreversible inhibitors.

We and others have shown that extracellular and intracellular CatB activity is a prerequisite for ECM turnover [10, 28, 29]. ECM provides an important physical barrier against tumor invasion and its proteolysis mediated by macrophages, tumor and endothelial cells creates a pathway for tumor and endothelial cells to migrate and invade the remodeled matrix [10-12, 30, 31]. All evaluated transformed and cancer cell lines degraded DQ-collagen IV intracellularly and extracellularly (Fig. 2A and 2B). Cell-permeable CatB inhibitor, CA-074Me, inhibited intracellular ECM degradation in MCF-10A neoT and U-87 MG cells but not in MMTV-PyMT and LPB-1 cells (Fig. 2C). This suggests that while in MCF-10A neoT and U-87 MG cells the intracellular ECM degradation is due, at least in part, to CatB activity, in the MMTV-PyMT and LPB-1 cell lines other proteases may be responsible for the intracellular breakdown of ECM proteins. On the other hand, CA-074 decreased the extracellular DQ-collagen IV degradation in all four cell lines, validating CatB as one of the key proteases involved in the extracellular breakdown of ECM. In line with this, nitroxoline reduced extracellular DQ-collagen IV degradation in all four cell lines tested (Fig. 2D). Furthermore, lower EC50 values for inhibition of extracellular versus intracellular DQ-collagen IV degradation suggest that nitroxoline is more efficient at inhibiting the extracellular rather than the intracellular arm of the ECM breakdown.

CatB has been proposed as one of the key proteases promoting tumor invasion. It was found equally relevant for epithelial, mesenchymal and glioma cell invasion that employ various migratory patterns, suggesting an universal mechanism for CatB in these processes [10, 29, 32-38]. Accordingly, siRNA-mediated knockdown, CA-074 and nitroxoline reduced tumor invasion as evaluated by xCELLigence system in real time in all evaluated cell lines (Fig. 3A). In addition to the 2D invasion assay, nitroxoline and CA-074 both reduced growth, sprouting and invasive cell migration in the 3D model of MCF-10A neoT, MMTV-PyMT and to a lesser extent of U-87 MG MCTS (Fig. 3B and 3C). On the other hand, LPB MCTS growth remained unaffected as they lacked the invasive corona around the original spheroid. Increasing literature reports show that in 3D spheroid models cancer cells acquire alternative phenotypes compared to monolayer cell culture due to increased cell-cell and cell-matrix interactions [39-41]. It is therefore possible that in U-87 MG and LBP cells other mechanisms, in addition to CatB, contribute to tumor invasion in the 3D experimental set up thus accounting for a lesser impact of nitroxoline on U-87 MG and LPB MCTS growth when compared to the 2D invasion model. Taken altogether, the xCELLigence and MCTS data demonstrate that by impairing ECM degradation nitroxoline is able to reduce tumor cell invasion in complex *in vitro* assays and indicate its feasibility for further *in vivo* evaluation as an antitumor agent.

The formation of tumor vasculature by angiogenesis is essential for tumors to grow beyond 1-2 mm^3^. CatB has been shown to promote angiogenesis by ECM breakdown and its role in angiogenesis has been validated in CatB knockout or overexpression mice [11, 15, 37-39]. Additionally, intense CatB staining of endothelial cells in glioblastomas correlated with poorer clinical outcomes confirming its role in tumor angiogenesis and suggesting its clinical application as a prognostic factor [42]. Nitroxoline attenuated endothelial tube formation of HMEC-1 and SVEC4-10 cells (Fig. 4). CA-074 displayed the largest inhibition on HMEC-1 cell tube formation which is in line with the CatB protein and activity levels being the highest of the three endothelial cell lines (Table 1). A recent study by Shim et al. [20] has shown that nitroxoline, through inhibition of MetAP2 and SIRT1, is able to induce senescence of endothelial cells, thereby inhibiting their proliferation and angiogenesis. For this reason, we included TNP-470 and EX-527, the inhibitors of MetAP2 and SIRT1, in our angiogenesis assay. TNP-470 failed to impair endothelial tube formation in all three cell lines tested, similarly as observed by Shim et al. [20] for HUVEC cells while EX-527 reduced tube formation of HMEC-1 but not of HUVEC or SVEC4-10 cells. The discrepancy between the two studies can be partially explained by differences in the experimental set-up. Nevertheless, nitroxoline may attenuate endothelial tube formation and angiogenesis by synergistic action on MetAP2, SIRT1 and CatB.

Anti-tumorigenic effects of nitroxoline were further evaluated in three independent tumor mouse models. Oral administration of nitroxoline attenuated the growth of LPB-induced tumors in C57Bl/6 mice (Fig. 5). Similar results were obtained with intraperitoneally applied CA-074. However, when nitroxoline and CA-074 were used in combination no additional decrease in tumor growth was observed, confirming that nitroxoline-mediated attenuation of tumor growth was due to CatB inhibition. As evident from tumor growth curves, nitroxoline treatment delayed the transition of tumors from avascular to vascular stages (Fig. 5A) confirming its anti-angiogenic properties.

Similar information was obtained in a FVB mouse model. Nitroxoline reduced the growth of MMTV-PyMT-induced tumors and impaired neovascularization compared to non-treated tumors (Fig. 6). Finally, when nitroxoline was administrated to transgenic PyMT mice that spontaneously form mammary carcinoma and metastases in the lung, it reduced the weight of mammary tumors, the number of metastases in the lung and their average size. Taken altogether, these results show that nitroxoline not only decreases tumor growth and angiogenesis but also impairs metastasis by inhibiting CatB activity.

In conclusion, we show that nitroxoline attenuates CatB-dependent extracellular ECM protein degradation and in this way abrogates processes of tumor invasion and angiogenesis in *in vitro* assays. Moreover, nitroxoline significantly abrogates tumor growth, angiogenesis and metastasis in various *in vivo* tumor mouse models regardless of its route of administration. This, together with its favorable pharmacokinetic/pharmacodynamic properties and lack of systemic toxicity, renders nitroxoline a new potential as anti-tumor drug that might also be used in combination with other cancer standards-of-care.

## MATERIALS AND METHODS

### Compounds

Nitroxoline, TNP-470 [43], EX-527 [44] and bafilomycin A1 were from Sigma-Aldrich (St. Louis, MO). CA-074 and CA-074Me were from PeptaNova (GmbH, Sandhausen, Germany). Chemical structures of nitroxoline, CA-074, CA-074Me, TNP-470 and EX-527 are provided in the Supporting Information (Supplementary Fig. 9).

### Cell lines

MCF-10A neoT, a c-Ha-ras oncogene transfected human breast epithelial cell line [40], and LPB fibrosarcoma cell line [41], a clonal derivate of methylcholanthrene-induced C57Bl/6 mouse sarcoma tumor were provided by Bonnie F. Sloane (Wayne State University, Detroit, MI) and Jean Jr. Belehradek (Institut Gustave Roussy, Villejuif, France), respectively. Primary MMTV-PyMT mammary carcinoma cells were isolated and cultured as described [42]. Human glioma cell line U-87 MG, human umbilical vein endothelial cell line HUVEC and SV40-transformed murine endothelial cell line SVEC4-10 were obtained from American Type Culture Collection (ATCC, Manassas, VA, USA). These cell lines were authenticated by ATCC using STR-PCR and were used within 6 months upon receipt. Human microvascular endothelial cells (HMEC-1) were obtained from Centers for Disease Control and Prevention (Atlanta, GA, USA). U-87 MG, MMTV-PyMT, HUVEC and SVEC4-10 cells were cultured in Advanced Dulbecco's Modified Eagle's Medium (DMEM; Invitrogen, Carlsbad, CA, USA) supplemented with 10% fetal bovine serum (FBS; HyClone, Little Chalfont, UK), 2 mM glutamine (Invitrogen) and antibiotics; LPB cells were cultured in Minimum Essential Medium (MEM; Invitrogen) supplemented with 10% FBS, 2 mM glutamine and antibiotics; MCF-10A neoT cells were cultured in DMEM/F12 (1:1) medium (Invitrogen) supplemented with 5% FBS, 1 μg/ml insulin (Sigma-Aldrich), 0.5 μg/ml hydrocortisone (Sigma-Aldrich), 50 ng/ml EGF (Sigma-Aldrich), 2 mM glutamine and antibiotics; HMEC-1 cells were cultured in MCDB 131 medium (Invitrogen) supplemented with 10 ng/ml EGF, 1 μg/ml hydrocortisone, 10% FBS, 10 mM L-glutamine and antibiotics. All cell lines were kept at 37°C in a humidified atmosphere containing 5% CO_2_.

### CatB protein and activity levels in whole-cell lysates and conditioned media

Conditioned medium was obtained by incubating the serum-free medium (SFM) with confluent cells for 24 h. Afterwards, media were concentrated and dialyzed twice against 100 mM acetate buffer (pH 5.5). Whole-cell lysates were prepared in lysis buffer (50 mM HEPES, 1 mM EDTA, 150 mM NaCl, 1% Triton X-100, pH 5.5). CatB protein levels were determined using ELISA as reported [43]. CatB activity was evaluated using a fluorogenic CatB endopeptidase substrate Z-Arg-Arg-AMC at 460 nm with excitation at 380 nm.

### Confocal microscopy

MCF-10A neoT cells (3×10^4^) were plated on glass coverslips in a 24-well plate and left to adhere overnight. Cells were transfected with 1μg/ml pPalmitoyl-mTurquoise2 plasmid (plasmid #36209, Addgene, Cambridge, MA) [52] for visualization of the membrane using Lipofectamine 2000 according to the manufacturer's protocol. After 24 h, cells were fixed with 5 % formalin (Sigma-Aldrich) for 20 min at room temperature (RT) and further permeabilized by 0.5 % Tween 20 in PBS for 10 min at RT. Non-specific staining was blocked with 3% bovine serum albumin (Sigma) in PBS for 1 h at RT and afterwards incubated with 3 μg/ml rabbit anti-CatB polyclonal antibody in blocking buffer for 1.5 h at RT. Next, cells were washed with PBS and incubated with Alexa Fluor 488 goat anti-rabbit secondary antibody (Invitrogen) for 1h at RT. After washing, a ProLong Antifade kit (Invitrogen) was used to mount coverslips on glass slides and the images were captured using a Zeiss LSM 710 confocal microscope (Carl Zeiss, Oberkochen, Germany) with ZEN 2011 image software.

### Western blot analysis

Equal amounts of protein were loaded and separated using 12% SDS-PAGE gels and transferred to a nitrocellulose membrane (GE Healthcare, Freiburg, Germany). Membranes were blocked with 5 % (w/v) non-fat dried milk powder in PBST (0.5 % Tween 20 in PBS) for 1 h at RT and afterwards incubated with sheep anti-CatB (1:2500) and mouse anti-β-actin (1:5000; Sigma-Aldrich) antibodies overnight at 4°C. Next, membranes were washed and incubated with HRP-conjugated rabbit anti-sheep secondary antibodies (1:3000) and goat anti-mouse IgG/IgM (1:1000; Millipore, Billerica, MA) antibodies 1h at RT for CatB and β-actin, respectively. Finally, membranes were washed and bands were visualized using SuperSignal West Dura Extended Duration Substrate (Thermo Scientific, Rockford, IL) or SuperSignal West Femto Maximum Sensitivity Substrate (Thermo Scientific) chemiluminiscence kit for CatB and β-actin, respectively using G:Box (Syngene, Cambrige, UK). Membranes were stripped with stripping buffer (100 mM 2-mercaptoethanol, 2% SDS and 62.5 mM Tris-HCl, pH 5.7) for 1h at 60°C. The band intensities were quantified using Gene Tools software (Syngene).

### CatB cell-surface expression

Cells (2×10^5^) were fixed with 10% formalin in PBS for 20 min on ice. After washing, cells were labelled with 10 μg/ml rabbit anti-CatB polyclonal antibody for 1h at RT. Afterwards, cells were incubated with Alexa Fluor 555 goat anti-rabbit secondary antibody (Invitrogen) for 1h at RT. Finally, cells were monitored for fluorescence using a FACSCalibur instrument (BD Biosciences, Franklin Lakes, NJ).

### DQ-collagen IV degradation

Quantification of intracellular and extracellular DQ-collagen IV (Invitrogen) degradation as well as its visualization were performed as reported [19].

### Cell viability assay

Possible cytotoxicity of the selected compounds on cell lines used in the invasion assays were evaluated using the xCELLigence System (Roche, Basel, Switzerland). The system monitors cellular events in real time by measuring the electrical impedance (expressed as cell index) generated by cells attached to the bottom of wells with integrated electrodes. 150 μl of MCF-10A neoT, U-87 MG, MMTV-PyMT (5×10^4^ cells/ml) and LPB (3.3×10^4^ cells/ml) cell suspension were seeded in the wells of an E-plate 16 (Roche) according to the manufacturer's instructions. After seeding, the CI was monitored every 15 min. After ∼10 h (MCF-10A neoT and MMTV-PyMT), 14 h (U-87 MG) or 24 h (LPB), when the cells were in their log phase of growth, 50 μl of the compound or 0.1% DMSO was added, and the experiment allowed to run for 72 h. Once every 24 h the medium was replaced with fresh medium containing the inhibitor or suitable control to prevent cell death due to medium depletion. Compounds and their concentrations were: nitroxoline (5 μM) and CA-074 (5 μM) for all cell lines other than MCF-10A neoT cell line, where nitroxoline was used at 2.5 μM. All measurements were performed in quadruplicate.

### Real time invasion assay

Tumor-cell invasion in real time were determined using the xCELLigence System as before [30]. Cells were serum-starved for 24 h before each experiment and the bottoms of CIM-16 plate wells (Roche) were coated with fibronectin (Calbiochem, Darmstadt, Germany). The upper compartments of the plate wells were coated with 20 μl of Matrigel (BD Biosciences) in SFM (5 mg/ml) and allowed to gel. After addition of 180 μl complete medium containing nitroxoline (2.5 or 5 μM), CA-074 (5 μM) or suitable control (0.05% DMSO) to lower compartments the plates were assembled and 60 μl of SFM with the compounds were added to the top wells. Afterwards, 80 μl of MCF-10A neoT, U-87 MG, MMTV-PyMT (3×10^4^ cells/well) and LPB (8×10^4^ cells/well) cell suspension were seeded in the top chambers. Impedance data, reported as cell index, was monitored for 72 h and the data were analyzed using the RTCA Software (Roche). For CatB silencing, cells were transfected with human (sc-29238; Santa Cruz Biotechnology, Dallas, TX or SI02663010; Qiagen, Limburg, Netherlands) or mouse CatB (sc-29933; Santa Cruz Biotechnology) or control siRNA (sc-37007; Santa Cruz Biotechnology) 6 h before the experiment, using Lipofectamine 2000 (Invitrogen) according to the manufacturer's protocol.

### Three-dimensional invasion assay

MCTS were prepared according to the hanging-drop method as before [44]. Formed MCTS were then transferred to wells of Lab-Tek™ Chambered Coverglass which had been coated with 70 μl of Matrigel (5 mg/ml) in SFM. Spheroids were covered with an additional 70 μl of Matrigel (5 mg/ml) in SFM and, after 20 min at 37°C, covered with 400 μl of complete medium. Nitroxoline (2.5 or 5 μM), CA-074 (5 μM), CA-074Me (5 μM) or DMSO (0.05 %) were added to the Matrigel and the medium and the growth of spheroids was monitored for up to three days by measuring the spheroid dimensions under a light microscope, using an ocular micrometer. Spheroid volume was calculated according to the equation: *V = (π × (spheroid length) × (spheroid width)^2^)/6*. In a separate experiment, CatB activity was assayed using a Magic Red^TM^ Cathepsin B Assay Kit according to manufacturer's instructions (Immunochemistry Technologies, Bloomington, MN). Images of tumor spheroids were obtained using an Olympus IX 81 motorized inverted microscope and Cell^R software (Olympus, Tokyo, Japan).

### Tube formation assay

Before each experiment cells were incubated for 24 h with corresponding compounds, then trypsinized and counted. Cells were then plated (SVEC4-10 – 2×10^4^, HUVEC – 1.9×10^4^, HMEC-1 – 1.3×10^4^ cells/well) on μ-Slide Angiogenesis (Ibidi, Martinsried, Germany) covered with 10 μl of undiluted Matrigel and incubated until the formation of tubular complexes. Tubular complexes were stained with Calcein AM according to manufacturer's instructions (Sigma-Aldrich). Images were captured with an Olympus DP72 CCD camera connected to an Olympus IX-70 inverted microscope. AxioVision program (Zeiss, Oberkochen, Germany) was used to convert raw images into binary masks of tubular complexes. The pixels above the threshold – Calcein positive – were given a value of 1 and all other pixels below the threshold – Calcein negative – were given a value of 0, creating a binary mask of the tubular networks that were quantified with the AngioQuant image analysis program [45]. The number, length and size of tubular complexes and the number of junctions were recorded.

### Mouse tumor models

Animal studies were carried out in accordance with EU guidelines and the permission from the Veterinary Administration of the Republic of Slovenia (permission number: #34401/42/2011/3 and #34401/15/2011/9). Mice were housed in a specific-pathogen-free animal colony at controlled temperature and humidity with 12h light/dark cycle. Food and water were provided *ad libitum*.

### LPB mouse fibrosarcoma tumor model

Experiments were performed in C57Bl/6 female mice, 8 – 12 weeks old. 1.2×10^6^ LPB fibrosarcoma cells in 0.9% NaCl, were injected into the right flank of each mouse. The control group was treated with drinking water only (n = 16). A 0.2 mg/ml solution of nitroxoline was given to mice *ad libitum* in their drinking water for the entire duration of the study (n = 13) or for 15 days only (n = 7). From the volume of consumed solution (4.0±0.7 ml/mouse/day), the mice received a nitroxoline dose of 40±7 mg/kg. CA-074 (10 mg/kg) was injected intraperitoneally every second day, alone (n = 7) or in combination with *ad libitum* nitroxoline in the drinking water (n = 6). Tumor growth was determined daily using a digital Vernier caliper, from the sixth day after the induction of tumors on. Tumor volume was calculated according to the following formula: *V = (tumor width^2^ × tumor length)/6*. From the tumor growth curves, the time required to reach a volume of 40 mm^3^ was determined. Tumor growth delay was determined as the difference in the time required for tumors to reach a volume of 40 mm^3^ between the experimental and the control group. The weight of the mice was followed as a general indicator of systemic toxicity.

### MMTV-PyMT mouse breast cancer model

FVB/N mice and a transgenic mouse strain expressing PyMT oncogene under the control of MMTV LTR promoter (FVB/N-TgN (MMTVPyVT)634-Mul) were used [27]. The orthotopic mouse model was established as described previously [46]. 5×10^5^ of primary MMTV-PyMT tumor cells in SFM were inoculated into the left inguinal mammary gland of the congenic recipient FVB mouse. Tumor bearing FVB mice were divided randomly into two groups. When tumor volume reached 125 mm^3^, mice were treated every second day, one group (n = 6) with vehicle as control, and the other (n = 6) with 40 mg/kg nitroxoline in 5% DMSO in peanut oil, intraperitoneally. Tumor volume was measured and calculated as mentioned above.

In a separate experiment, transgenic PyMT mice were divided randomly into two groups at the age of 11 weeks. They were treated every second day, one group (n = 10) was injected intraperitoneally with vehicle, and the other group (n = 14) with 40 mg/kg nitroxoline in 5% DMSO in peanut oil. Mice were sacrificed at 14 weeks of age; mammary tumor tissues were collected and weighed. Lungs were dissected and processed for histomorphometric analysis. Tumor vascularization, the number and the average size of lung metastasis in PyMT mice was determined as reported [34].

### Statistical analysis

Statistical significance of differences between groups of data was evaluated using the two-sided Student's t-test using SigmaPlot Software (Systat Software, San Jose, CA) unless stated otherwise.

## SUPPLEMENTARY FIGURES


