# Supplementary material for: Nitroxoline impairs tumor progression in vitro and in vivo by regulating cathepsin B activity
Source: Oncotarget. 2015 Mar 30;6(22):19027–42. doi: 10.18632/oncotarget.3699 (PMC4662473; doi:10.18632/oncotarget.3699)
Supplement: Supplementary file 1 [file oncotarget-06-19027-s001.pdf]

Nitroxoline impairs tumor progression *in vitro* and *in vivo* by regulating cathepsin B activity

Supplementary Material

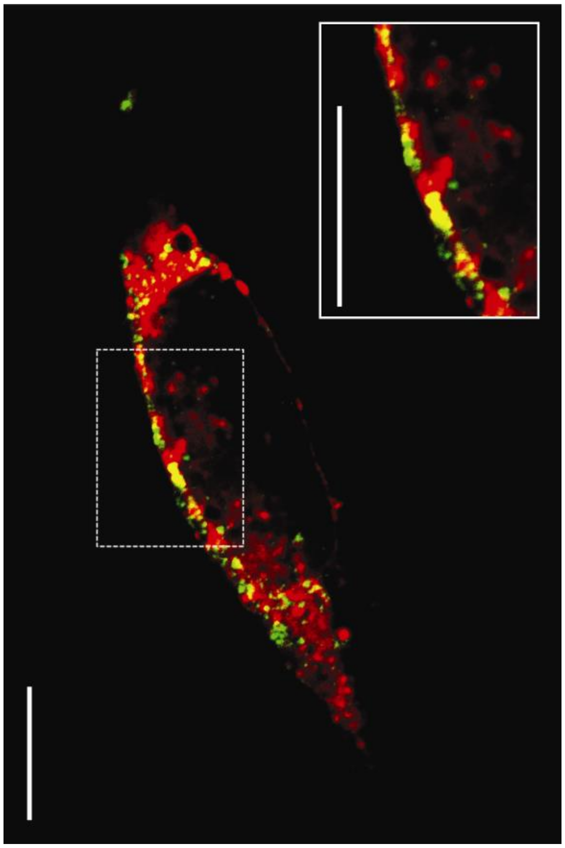

Supplementary Figure 1: Cathepsin B cell surface expression in MCF-10A neoT cells as evaluated by confocal microscopy. MCF-10A neoT cells (3×10^4) were grown on coverslips and transfected with 1µg/ml pPalmitoyl-mTurquoise2 plasmid for visualization of the membrane. Cathepsin B staining was performed using a rabbit anti-cathepsin B polyclonal antibody followed by Alexa Fluor 488 goat anti-rabbit secondary antibody. Cathepsin B and plasma membrane are visualized as green and red fluorescence, respectively and colocalization is shown

in yellow. Images shown are representative of three independent experiments performed. Scale bar, 10 μm.

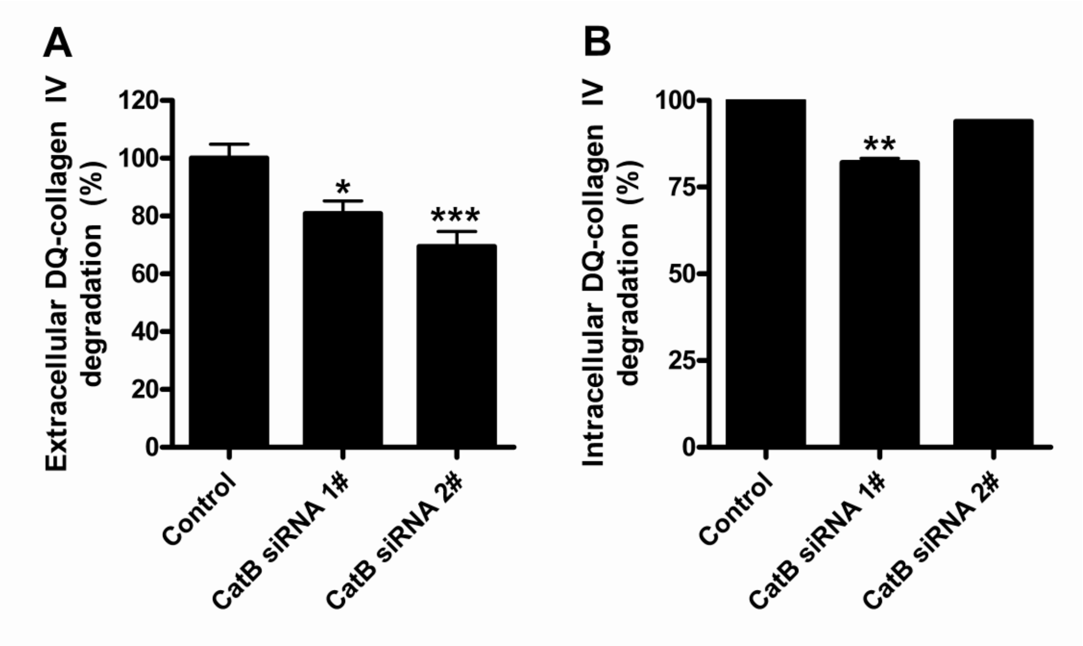

Supplementary Figure 2: Cathepsin B knockdown reduces extracellular and intracellular DQ-collagen IV degradation by MCF-10A neoT cells. MCF-10A neoT cells (2 and 3×10^4/well, respectively) were seeded into wells of a 96- and 24-well plate, respectively and allowed to attach overnight. Cells were then treated with human cathepsin B-specific siRNA (siRNA 1# and 2#) or control siRNA with a scrambled sequence, according to manufacturer's protocol. Three days after transfection, (A) extracellular and (B) intracellular DQ-collagen IV degradation were evaluated according to the protocol described in the main text. All data are presented as means ± SEM, n = 3 (* $p<0.05$, ** $p< 0.01$).

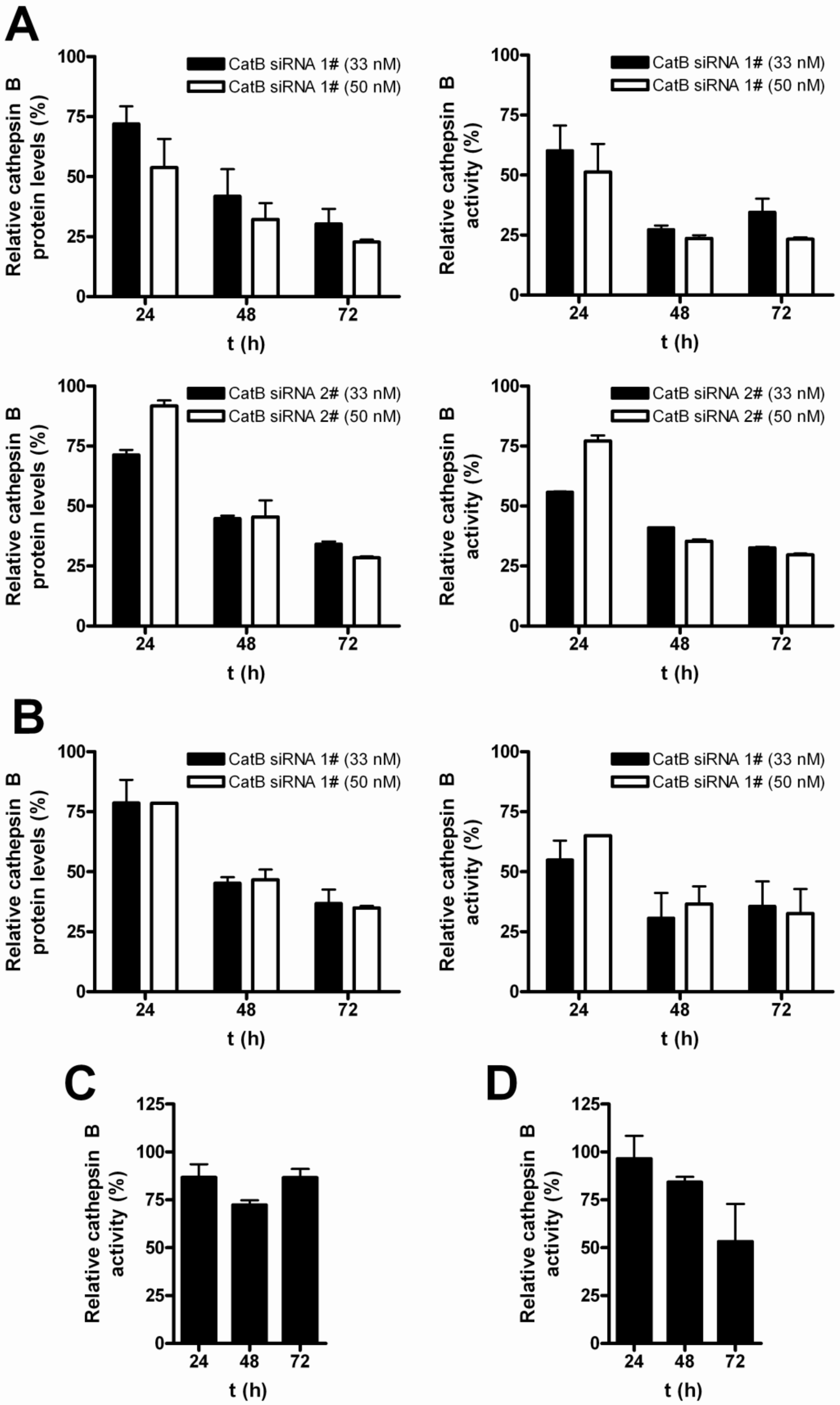
A
CatB siRNA 1# (33 nM)
CatB siRNA 1# (50 nM)
Relative cathepsin B protein levels (%)
Relative cathepsin B activity (%)
CatB siRNA 2# (33 nM)
CatB siRNA 2# (50 nM)
B
C
D
t (h)
24
48
72

Supplementary Figure 3: Cathepsin B protein and activity levels in cathepsin B knockdown MCF-10A neoT, U-87 MG, MMTV-PyMT and LPB cells. (A) 8×10^4 of MCF-10A neoT, (B) U-87 MG, (C) MMTV-PyMT and (D) LPB cells were plated in a 12-well plate and left to adhere overnight in an antibiotic-free medium. The cells were then transfected with 33 nM or 50 nM (MCF-10A neoT and U-87 MG) and 66 nM (MMTV-PyMT and LPB) of siRNA targeting human (siRNA 1# or siRNA 2#) or mouse cathepsin B or control siRNA using Lipofectamine 2000 according to the instructions of the manufacturer. The medium was replaced with fresh medium 6 h after transfection and cells harvested and lysed at 24, 48 and 72 h. Cathepsin B protein and activity levels in prepared lysates were determined using cathepsin B-specific ELISA and cathepsin B-specific fluorogenic substrate Z-Arg-Arg-AMC. All data are presented as means ± SEM, n = 3.

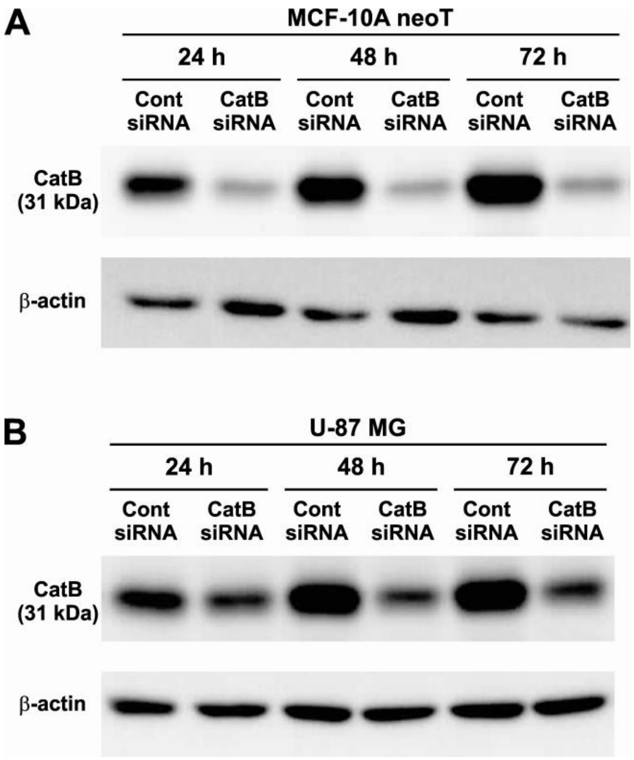

Supplementary Figure 4: Cathepsin B knockdown in MCF-10A neoT and U-87 MG cells as assessed with western blot. (A) 8×10^4 of MCF-10A neoT and (B) U-87 MG cells were plated in a 12-well plate and left to adhere overnight in an antibiotic-free medium. The cells were then transfected with 33 nM of siRNA targeting human cathepsin B or control siRNA using Lipofectamine 2000 according to the instructions of the manufacturer. The medium was replaced with fresh medium 6 h after transfection and cells were harvested and lysed at 24, 48 and 72 h. Cathepsin B protein levels in whole cell lysates were analyzed with western blot analysis. siRNA knockdown reduced cathepsin B protein levels by 82, 90 and 83% in MCF-10A neoT cells and by 35, 62 and 62% in U-87 MG cells at 24, 48 and 72 h. Shown are representative images of three independent experiments performed.

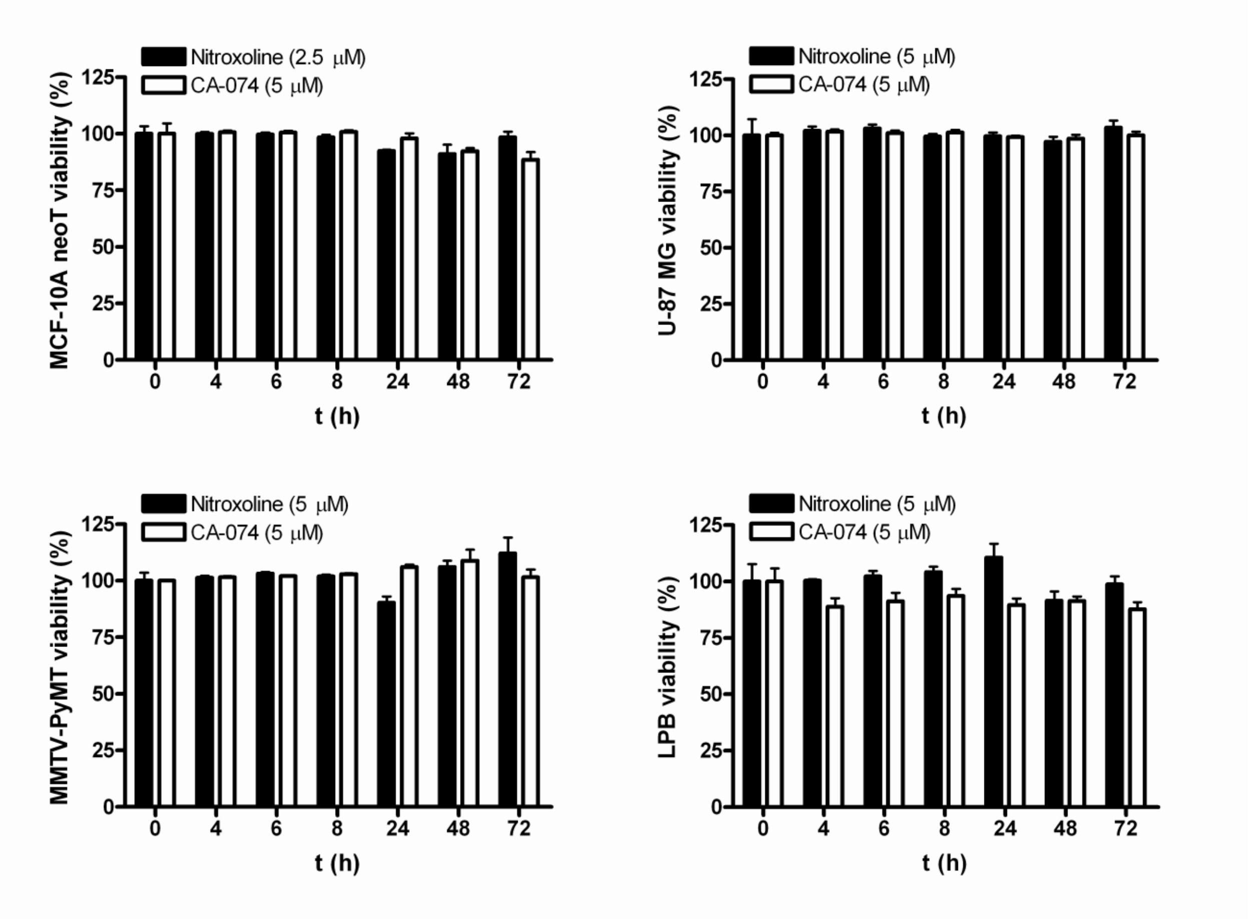

Supplementary Figure 5: Cytotoxicity of nitroxoline and CA-074 against MCF-10A neoT, U-87 MG, MMTV-PyMT and LPB cell lines, as assessed by xCELLigence. 150 μl of MCF-10A neoT, U-87 MG, MMTV-PyMT (5×10^4 cells/ml) and LPB (3.3×10^4 cells/ml) cell suspensions were seeded in the wells of an E-plate 16. CI was then monitored every 15 min. After ~10 h (MCF-10A neoT and MMTV-PyMT), 14 h (U-87 MG) or 24 h (LPB), 50 μL of the compound or suitable control (0.1% DMSO) was added, and the experiment allowed to run for 72 h. All data are presented as means ± SEM, n=4.

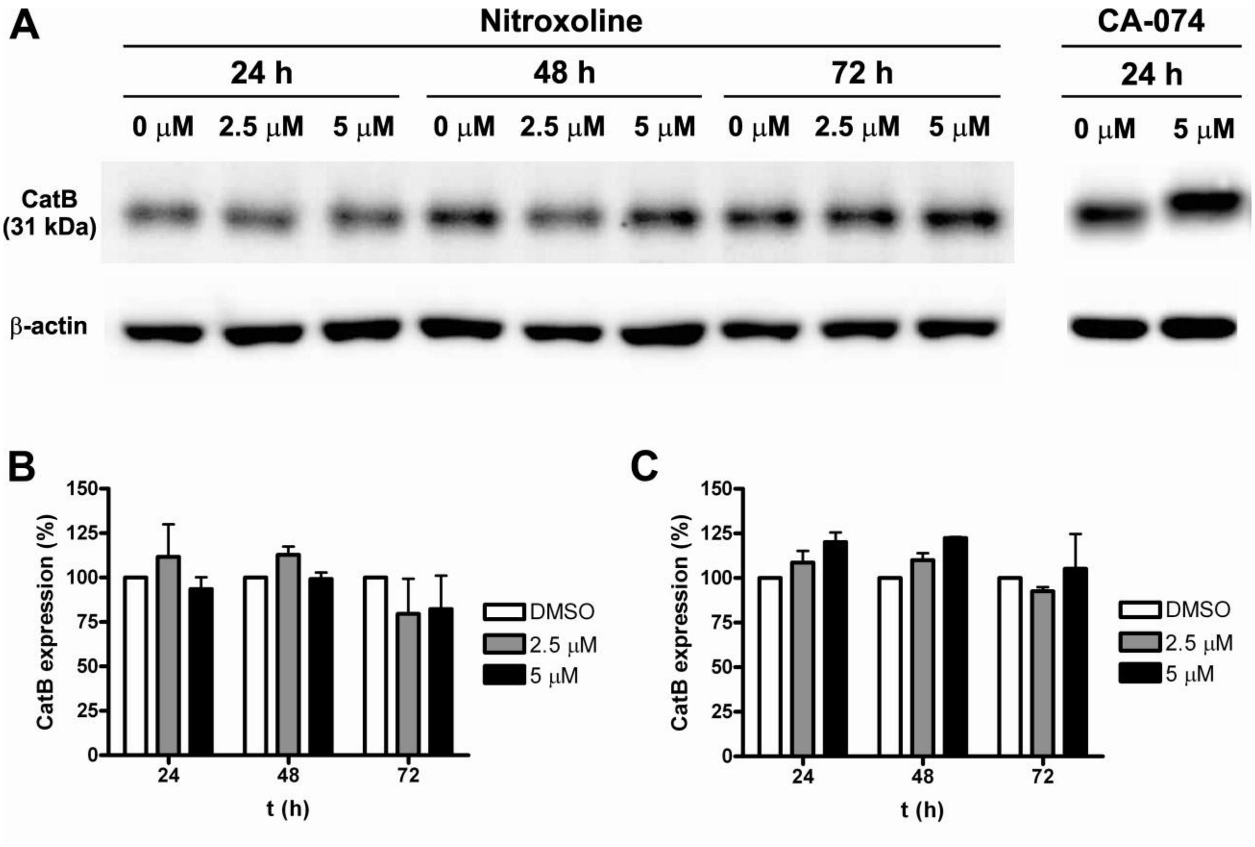

Supplementary Figure 6: Expression of cathepsin B in presence of nitroxoline evaluated by western blot analysis and ELISA. MCF-10A neoT cells (8×10^4) were plated in the wells of a 12-well plate and left to adhere overnight. Cells were then treated with nitroxoline (2.5 or 5 µM) or 0.05% DMSO as control. After 24, 48 and 72 h whole cell lysates were prepared in lysis buffer supplemented with protease inhibitor cocktail (Thermo Scientific). Total protein concentration was determined with the Lowry method and cathepsin B expression in lysate samples was evaluated with (A and B) western blot analysis and (C) ELISA. All data are presented as means ± SEM, n=3.

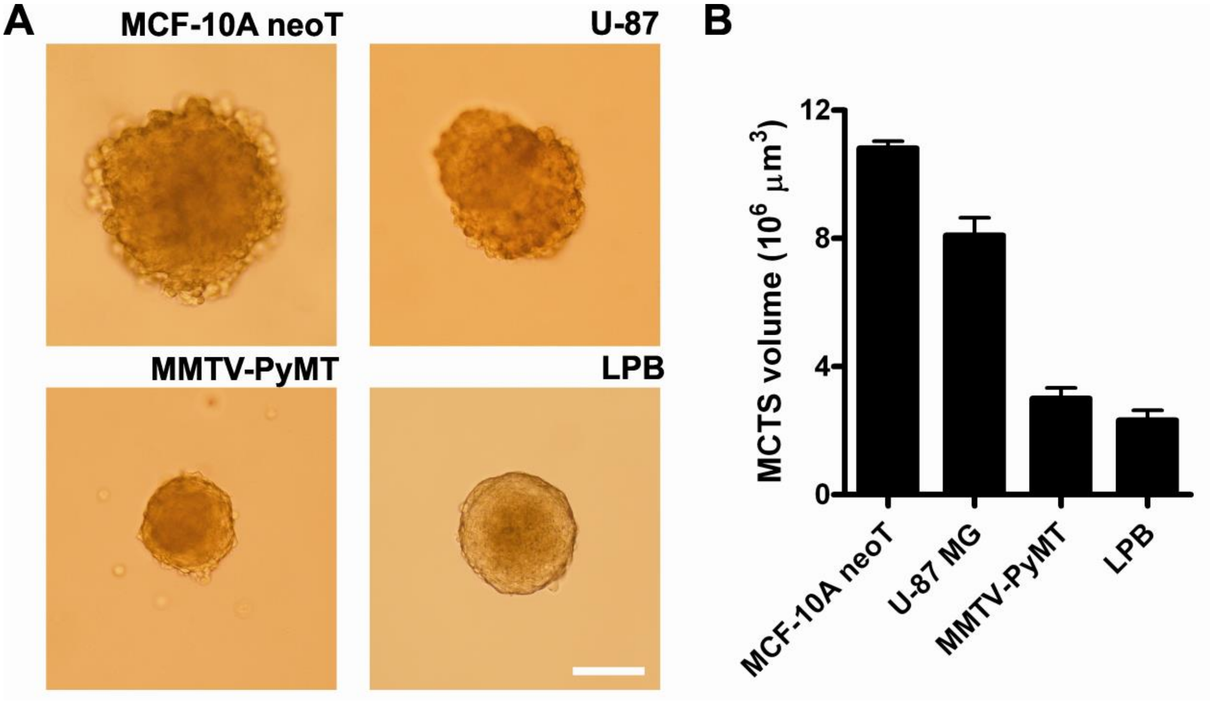

Supplementary Figure 7: Preparation of multicellular tumor spheroids (MCTS) of reproducible shape and size using the hanging drop method. (A) 20 µl drops of MCF-10A neoT (250 cells/drop), U-87 MG (150 cells/drop), MMTV-PyMT (250 cells/drop) and LPB (500 cells/drop) cell suspension were placed on the lids of petri dishes which were then inverted over 10 ml of sterile water. After 6 (MCF-10A neoT), 5 (U-87 MG) and 4 days (MMTV-PyMT and LPB), representative pictures of MCTS were taken with a CX21FS1 Olympus microscope equipped with a Olympus Camedia C-7070 Wide Zoom camera. (B) The MCTS dimensions were measured under a light microscope equipped with an ocular micrometer. Spheroid volume was calculated according to the equation: $V = (\pi \times (spheroid\ length) \times (spheroid\ width)^2)/6$. Data are presented as means ± STDEV, n > 5. Scale bar, 100 µm.

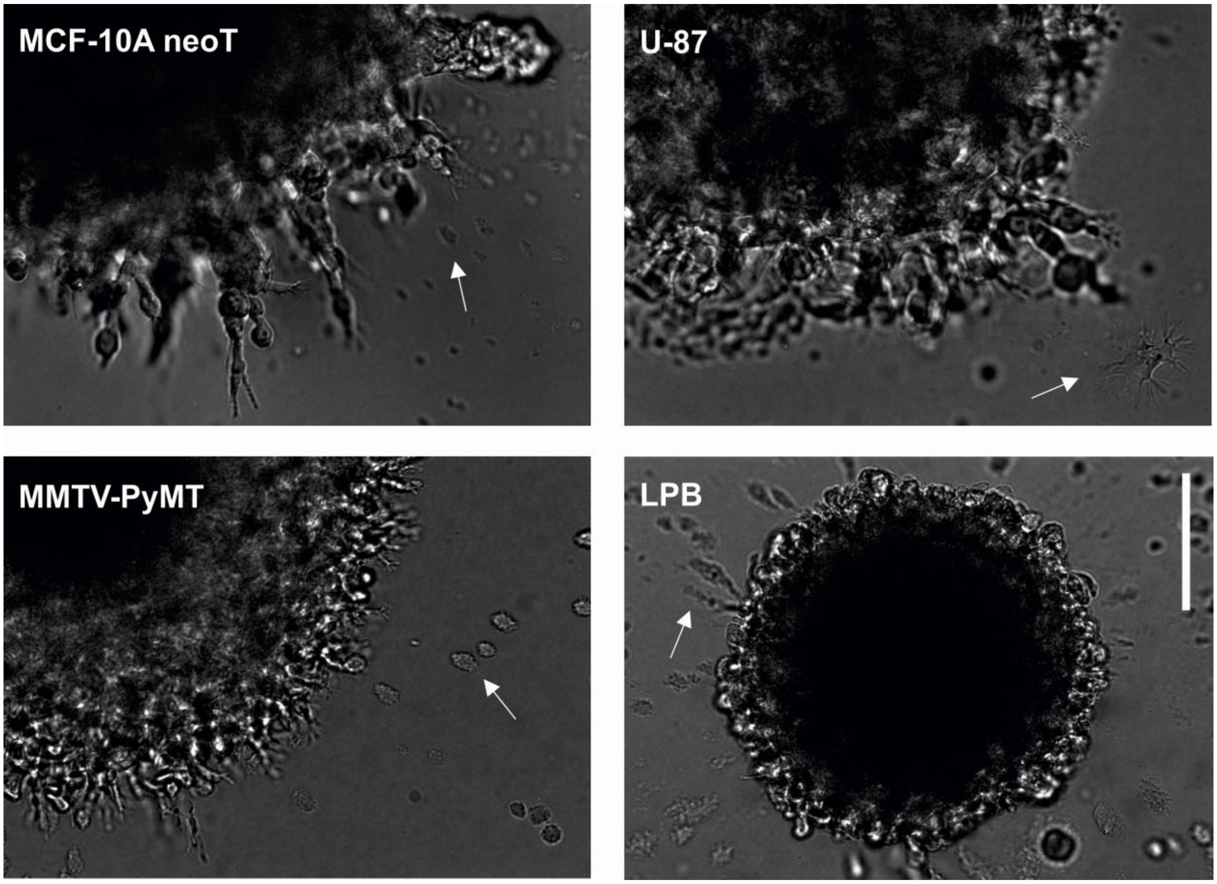

Supplementary Figure 8: Individual cells migrating away from the original MCTS (white arrows). Scale bar, 100 µm.

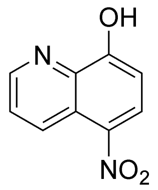

Nitroxoline

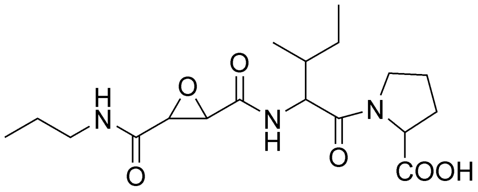

CA-074

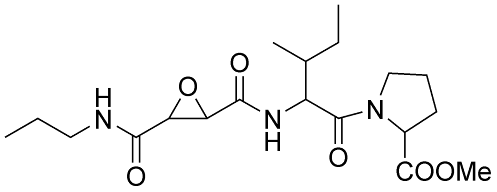

CA-074Me

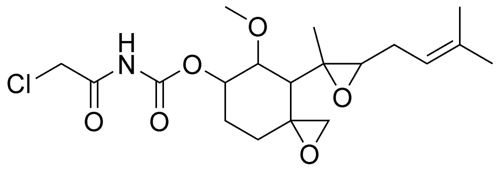

TNP-470

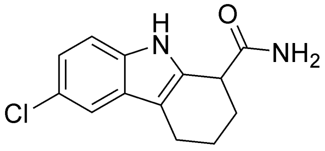

EX-527

Supplementary Figure 9: Chemical structures of nitroxoline, CA-074, CA-074Me, TNP-470 and EX-527.
